# The impact of upright posture on left ventricular deformation in athletes

**DOI:** 10.1007/s10554-023-02820-2

**Published:** 2023-03-04

**Authors:** J. Kandels, M. Metze, A. Hagendorff, R. P. Marshall, P. Hepp, U. Laufs, S. Stöbe

**Affiliations:** 1 Klinik und Poliklinik für Kardiologie, Universitätsklinikum Leipzig, Liebigstr. 20, 04103 Leipzig, Germany; 2 RasenBallsport Leipzig GmbH, Cottaweg 3, 04177 Leipzig, Germany; 3 Department of Orthopedic and Trauma Surgery, Martin-Luther-University Halle-Wittenberg, 06120 Halle, Germany; 4 Klinik und Poliklinik für Orthopädie, Unfallchirurgie und Plastische Chirurgie, Universitätsklinikum Leipzig, Leipzig, Germany

**Keywords:** Echocardiography, Athletes, Upright posture, Deformation, Longitudinal strain, Work index

## Abstract

Besides LV ejection fraction (LVEF), global longitudinal strain (GLS) and global myocardial work index (GWI) are increasingly important for the echocardiographic assessment of left ventricular (LV) function in athletes. Since exercise testing is frequently performed on a treadmill, we investigated the impact of upright posture on GLS and GWI. In 50 male athletes (mean age 25.7 ± 7.3 years) transthoracic echocardiography (TTE) and simultaneous blood pressure measurements were performed in upright and left lateral position. LVEF (59.7 ± 5.3% vs. 61.1 ± 5.5%; P = 0.197) was not affected by athletes’ position, whereas GLS (− 11.9 ± 2.3% vs. − 18.1 ± 2.1%; *P* < 0.001) and GWI (1284 ± 283 mmHg% vs. 1882 ± 247 mmHg%; *P* < 0.001) were lower in upright posture. Longitudinal strain was most frequently reduced in upright posture in the mid-basal inferior, and/or posterolateral segments. Upright posture has a significant impact on LV deformation with lower GLS, GWI and regional LV strain in upright position. These findings need to be considered when performing echocardiography in athletes.

**Graphical Abstract:**

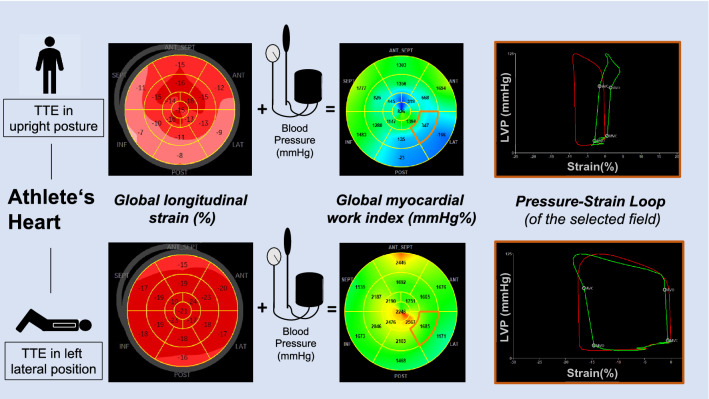

## Introduction

To prevent life-threatening events (e.g. sudden cardiac death) in competitive athletes with unknown cardiovascular disease, transthoracic echocardiography (TTE) is recommended by major sport associations [1].

Although left ventricular (LV) ejection fraction (LVEF) is mainly used to characterize LV systolic function, LV global longitudinal strain (GLS) has shown to be more sensitive in the detection of subclinical LV dysfunction than LVEF [, , , 2–5]. Further, GLS assessed by speckle tracking analysis has shown lower intra- and interobserver variabilities compared to LVEF measurements [6]. In healthy individuals GLS varies from − 22% to − 18% [7], whereas the inter-vendor variability of GLS analyses need to be considered [, 7, 8].

While GLS is dependent on pre- and afterload conditions [9], global myocardial work index (GWI) is a modern echocardiographic parameter which has been shown to be afterload independent with respect to myocardial deformation and contractile function [, 10, 11]. Based on GWI, further parameters such as global myocardial work efficiency (GWE) defined as the percentage ratio of constructive work (GCW) to the sum of GCW and wasted work (GWW) enable the analysis of LV function irrespective of afterload conditions [11]. Normal values of GWI and GCW vary from 1900 to 2100 mmHg % to 2200–2400 mmHg %. Reference values of GWW is defined from 73 to 87 mmHg%, and mean GWE is about 96% [12].

TTE at rest is usually performed in left lateral position. In competitive athletes it is not uncommon that exercise testing is performed in upright posture—especially when testing on a treadmill [13]. While the impact of body position on physiological parameters (e.g. blood pressure, heart rate) is well described [14], the impact of upright posture on LV deformation parameters has not been described yet.

The objective of this study was to analyze the impact of upright posture on echocardiographic parameters of LV deformation in healthy athletes. We hypothesized that upright posture has no significant impact on LV deformation.

## Methods

**Fig. 1:**
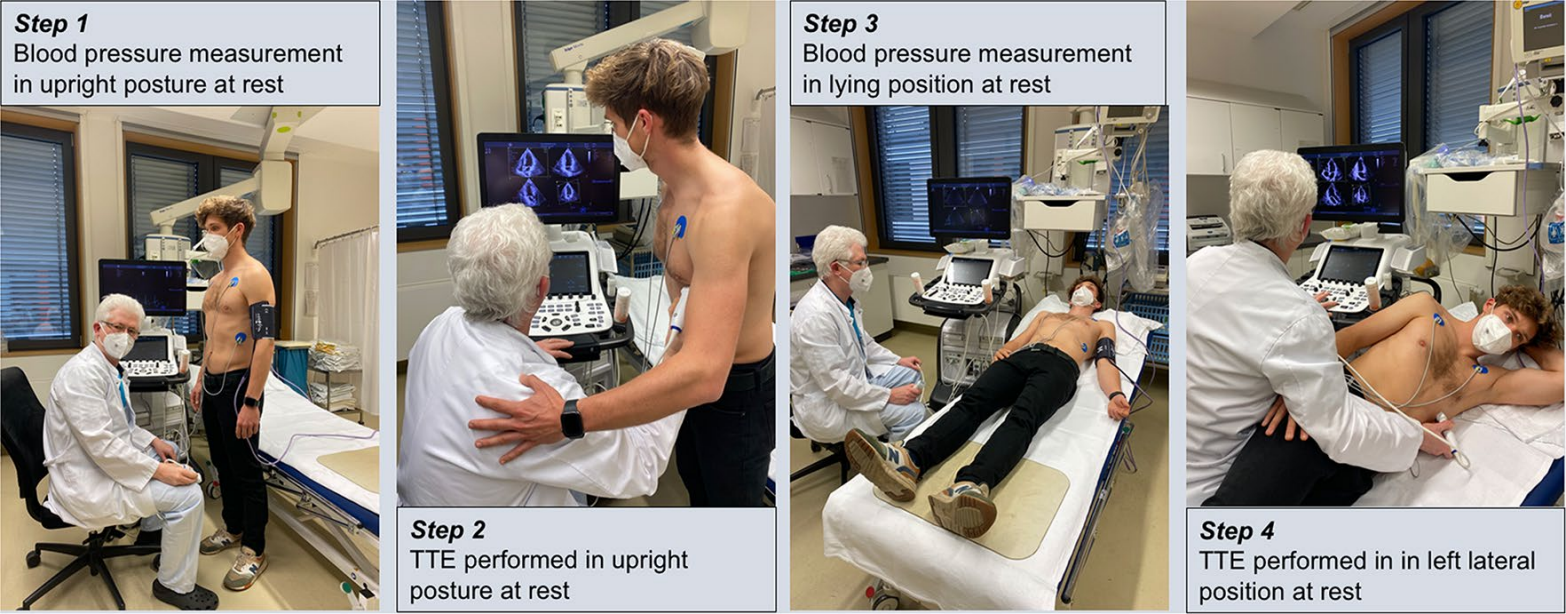
The diagnostic algorithm. *TTE *Transthoracic echocardiography

In this study 50 male athletes who underwent TTE during pre-participation screening at the University Hospital Leipzig between March 2018 and August 2021, were included. All athletes were practicing sports at a competitive level with more than 20 h of training per week. They were examined in upright posture and left lateral position and provided informed consent after full explanation of the purpose and order of all procedures (Fig. 1). The study was conducted in accordance with the Declaration of Helsinki and approved by the ethical committee of the University of Leipzig (**073/18-ek**).

TTE was performed according to a standardized protocol using a Vivid e9 or Vivid e95 ultrasound system with a 4Vc phased array probe (GE Healthcare Vingmed Ultrasound AS, Horten, Norway). All examinations were documented by a board certified cardiologist. Data sets were analyzed by post-processing analyses using the EchoPac software (Version 204, GE Healthcare Vingmed Ultrasound AS, Horten, Norway). Systolic and diastolic blood pressure was measured in all patients after five minutes resting in upright position and in left lateral position.

### Conventional echocardiographic parameters

The left ventricular outflow tract diameter (D_LVOT_) was determined in the parasternal long axis view in the left lateral position. Relative wall thickness (RWT) was calculated by twice of the LV posterior wall diameter (LVPWD) divided by LV end-diastolic diameter (LVEDD) measured by anatomical M-Mode in parasternal short axis views verified by biplane scanning. LV longitudinal axis diameter (LVLD) was assessed in the apical long axis view by measuring the distance from the apex to the center of the closed mitral valve at end-diastole. LV mass (LVM) was calculated by the Deveraux formula and was indexed to the body surface area (LVMi). Normal ranges of LVMi were defined < 115 g/m^2^ (males) [15].

The LVOT velocity time integral (VTI_LVOT_) was measured by pulsed waved (PW-) Doppler in the apical long axis view with the sample volume exactly at D_LVOT_ measurement position. LV stroke volume (SV_Doppler_) was calculated by the cross-sectional area of the LVOT multiplicated by VTI_LVOT_. The LVEF, LV end-diastolic (LVEDV) and end-systolic (LVESV) volumes and LVSV_biplane_ were assessed by LV biplane planimetry by the modified Simpson’s rule in the apical 2- and 4-chamber view [15]. Regarding both approaches the LVSV was indexed to the body surface area (LVSVi). Left atrial volume index (LAVi) was determined according to current recommendations [15].

### Deformation imaging

Deformation imaging was based on speckle tracking analyses using standardized apical views differing by 60° (long axis view, 2-chamber and 4-chamber view) according to current recommendations [16]. The beginning of the QRS complex was set as the reference point, whereas the end-systole was defined by the end of the ejection period obtained from PW Doppler measurements in the LVOT. The region of interest (ROI) of the myocardial tracking area was adjusted to the endocardial and epicardial border. LV longitudinal deformation was assessed to detect regional deformation abnormalities using the 18-segment model comprising all apical views [15]. Regional longitudinal strain values were determined for each LV segment, GLS of > − 16.7% was considered to be normal [17]. GWI, GCW, GWW and GWE of the LV were calculated by post-processing analyses taking systolic and diastolic blood pressure into account.

### Statistical analysis

All statistical analyses were performed using SPSS Statistics version 28.0 (IBM, Armonk, NY). Normal distribution was tested using Kolmogorov–Smirnov. Continuous variables were expressed as mean value ± standard deviation (SD) and were compared between groups using Student’s t-test. All categorical variables were expressed as numbers with their percentages (%) and compared using chi-squared or Fisher exact test, as appropriate. Linear regression and Pearson’s r were applied to evaluate association between two linear variables. Data comparisons between more than two groups were performed by one-way Analysis of Variance (ANOVA). A *P* value < 0.05 was considered statistically significant.

Kappa coefficient (κ) was used to assess intra- and interobserver variabilities in 20 patients under the same conditions. The second investigator was unaware of the results of the first examination.

## Results

### Baseline demographic characteristics

In 50 male athletes (25.7 ± 7.3 years) systolic (128.3 ± 8.3 mmHg vs. 125.3 ± 9.9 mmHg; *P* = 0.104) and diastolic blood pressure values (74.3 ± 6.8 mmHg vs. 72.9 ± 12.2 mmHg; *P* = 0.482) did not differ in upright posture compared to left lateral position. The heart rate was higher in upright posture compared to left lateral position (79.1 ± 13.9 /min vs. 61.0 ± 10.1 /min; *P* < 0.001). Further demographic data are presented in Table 1.

**Table 1:** Baseline demographic characteristics

Variables (n = 50)	
Age (years)	25.7 ± 7.3
Sex (%)	100
Weight (kg)	83.9 ± 12.2
Height (cm)	186.2 ± 6.5
BSA (m^2^)	2.08 ± 0.17
BMI (kg/m^2^)	24.1 ± 2.3

*BSA* body surface area, *BMI* body mass index

### Conventional echocardiography

Whereas LVMi was not affected by position changing (98.3 ± 21.0 g/m^2^ vs. 106.0 ± 16.80 g/m^2^ *P* = 0.072), RWT was higher in upright posture compared to left lateral position (0.40 ± 0.05 vs. 0.35 ± 0.05; p < 0.001). LVLD, LVEDD and LVESD were lower in upright posture compared to left lateral position (Table 2). Stroke volume measurement LVSV_Doppler_ (65.3 ± 14.2 ml vs. 94.7 ± 15.8 ml; *P* < 0.001) and LVSV_biplane_ (69.1 ± 16.3 ml vs. 95.1 ml ± 16.0 ml; *P* < 0.001) decreased in upright compared to left lateral position.

**Table 2:** Hemodynamic and conventional echocardiographic parameters

Variables	Upright posture	Left lateral position	p value
sBP (mmHg)	128.3 ± 8.3	125.3 ± 9.9	0.104
dBP (mmHg)	74.3 ± 6.8	72.9 ± 12.2	0.482
HR (1/min)	79.1 ± 13.9	61.0 ± 10.1	< 0.001*
IVSD (mm)	9.9 ± 1.2	9.7 ± 1.4	0.299
LVPWD (mm)	9.6 ± 1.1	9.3 ± 1.2	0.383
LVEDD (mm)	48.2 ± 4.2	54.8 ± 5.0	< 0.001*
LVESD (mm)	31.9 ± 3.7	34.8 ± 4.7	< 0.001*
LVMi (g/m^2^)	98.3 ± 21.0	106.0 ± 16.8	0.072
RWT	0.40 ± 0.05	0.35 ± 0.05	< 0.001*
LVLD (mm)	8.8 ± 0.8	9.9 ± 0.8	< 0.001*
LVEDV biplane (ml)	117.4 ± 29.5	157.9 ± 30.8	< 0.001*
LVESV biplane (ml)	48.3 ± 15.3	62.7 ± 19.1	< 0.001*
LVSV biplane (ml)	69.1 ± 16.3	95.1 ± 16.0	< 0.001*
LVSVi biplane (ml/m^2^)	33.2 ± 7.0	45.7 ± 6.4	< 0.001*
LVSV Doppler (ml)	65.3 ± 14.2	94.7 ± 15.8	< 0.001*
LVSVi Doppler (ml/m^2^)	31.2 ± 6.0	45.4 ± 7.2	< 0.001*
LAVi (ml/m^2^)	7.7 ± 4.6	18.2 ± 5.8	< 0.001*

*significant difference (p < 0.05). sBP systolic blood pressure, *dBP* diastolic blood pressure, *HR* heart rate, *IVSD* Interventricular septum diameter, *PWD* Posterior wall diameter, *LVEDD* left ventricular end-diastolic diameter, *LVESD* left ventricular end-systolic diameter, *LVMi* left ventricular mass index, *RWT* relative wall thickness, *LVLD* left ventricular longitudinal axis diameter, *LVEDV* left ventricular end-diastolic volume, *LVESV* left ventricular end-systolic volume, *LVSV* left ventricular stroke volume, *LVSVi* left ventricular stroke volume index, *LAVi* left atrial volume index

### Parameters of left ventricular systolic function

**Fig. 2:**
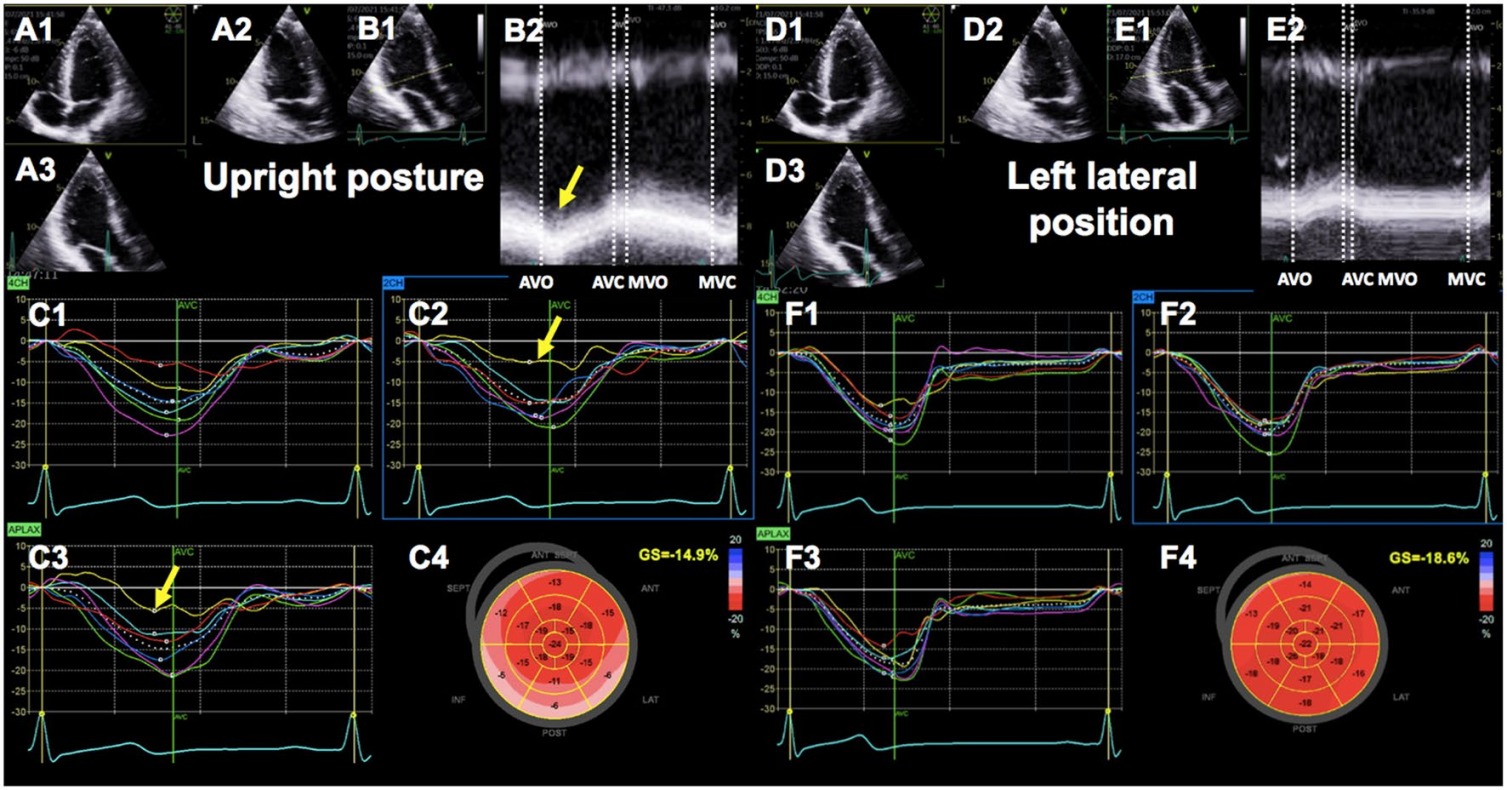
Global longitudinal strain measured by 2D echocardiography in upright (**A-C**) and left lateral position in the same athlete (**D-F**). Apical long axis, 2- and 4-chamber view **(A1-A3, D1-D3)** with anatomical M-Mode in the apical long axis view (**B1-B2, E1-E2**). Strain curves of all three apical views (**C1-C3, F1-F3**) with the corresponding bull’s eye (**C4**, **F4**). The yellow arrow indicates changes in left ventricular wall motion in comparison to left lateral position. *AVO *Aortic valve opening, *AVC *Aortic valve closing, *MVO *Mitral valve opening, *MVC *Mitral valve closing

**Fig. 3:**
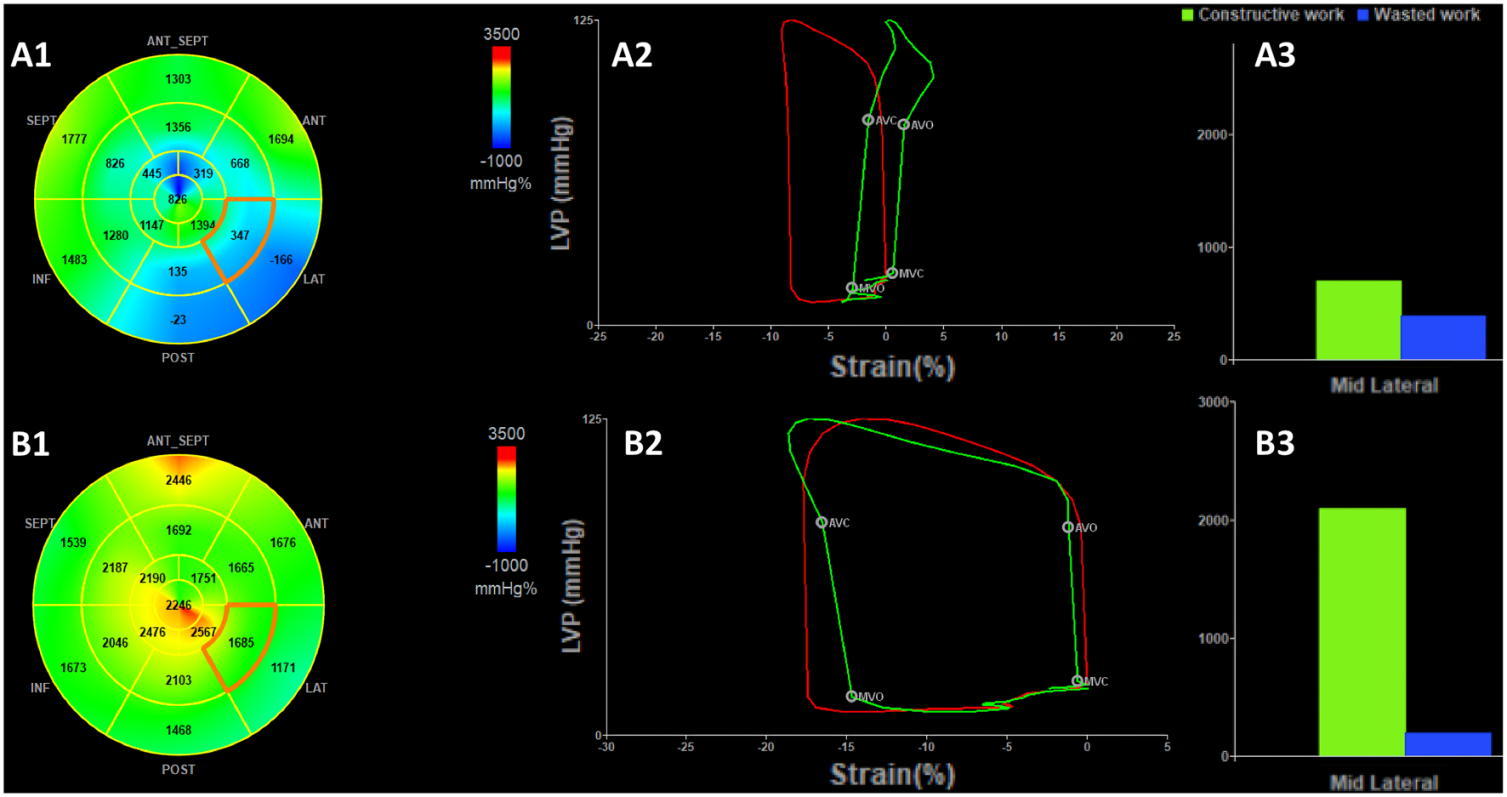
Assessment of global myocardial work index in upright (**A**) and left lateral position (**B**). Bull’s eye of global myocardial work index (**A1**, **B1**) with the pressure-strain-loop of the mid-lateral segment of the left ventricle (**A2**, **B2**). Comparison of constructive and wasted work in the mid-lateral segment of the left ventricle (**A3**, **B3**)

LVEF did not differ significantly between upright and left lateral position (61.1 ± 5.5% vs. 59.7 ± 5.3%; *P* = 0.197, Table 3). GLS, GWI, GCW and GWE were significant lower in upright posture compared to left lateral position (Table 3). GWW was significant higher in upright posture compared to left lateral position. In all athletes a reduction of regional longitudinal strain was observed in upright posture in the inferior and/or posterolateral LV segments (Figs. 2, 3). According to longitudinal strain, a regional reduction of the myocardial work index was observed in the inferior and/or posterolateral segments.

**Table 3:** Echocardiographic parameters of left ventricular systolic function

Variables	Upright posture	Left lateral position	p value
EF (%)	59.7 ± 5.3	61.1 ± 5.5	0.197
CO (l/min)	4.72 ± 1.12	5.28 ± 0.98	< 0.001*
CI ((l/min)/m^2^)	2.26 ± 0.49	2.54 ± 0.43	0.003*
GLS (%)	−13.5 ± 2.6	−18.5 ± 1.8	< 0.001*
GWI (mmHg%)	1284.6 ± 282.9	1882.4 ± 246.6	< 0.001*
GCW (mmHg%)	1734.2 ± 249.2	2073.1 ± 254.4	< 0.001*
GWW (mmHg%)	241.9 ± 149.7	76.5 ± 31.2	< 0.001*
GWE (%)	86.9 ± 6.7	95.8 ± 1.5	< 0.001*

*significant difference (p < 0.05). EF ejection fraction, *CO* cardiac output, *CI* cardiac index, *LAVi* left atrial volume index, *GLS* Global longitudinal strain, *GWI* Global work index, *GCW* Global constructive work, *GWW* Global wasted work, *GWE* Global work efficiency

### Intra- and Interobserver variability

Intraobserver variability showed high agreement for GLS in upright posture (κ = 0.81; z = 4.1, *P* < 0.001) as well as in left lateral position (κ = 0.88; z = 4.43, *P* < 0.001). Interobserver variability between two investigators showed equally good agreement for GLS in upright posture (κ = 0.74; z = 4.27, *P* < 0.001) as well as in left lateral position (κ = 0.86; z = 5.11; *P* < 0.001). Intra- and interobserver variabilities for the remaining conventional echocardiographic measurements consistently showed good agreement as well.

## Discussion

*The main findings of the present study are the detection of significant differences of LV morphology and function between upright and left lateral position determined by TTE at rest. These results are based on* ***(1)*** *smaller LV volumes and shorter longitudinal LV axis,* ***(2)*** *significantly reduced GLS—mainly due to regional deformation differences in the basal inferior and posterolateral LV segments, and* ***(3)*** *lower GWI in upright position.*

Changes of LV volumes in relation to body position have been reported by Nixon et al. observing a decrease of 30–45% of LVSV as well as an increase of 25–45% in heart rate in healthy young men after mobilization from supine to upright position [18]. Upright position increased long axis shortening, LVEDD and LVESD according to Sundblad and Wranne as shown in healthy subjects [19]. Goodman et al. analyzed left ventricular function after leg cycling and reported an increase of LVEDV, possibly due to increased venous return [20]. These observations were in line with the results of our study and could be physiologically explained by a fast response of the smooth muscles of the venous and arterial vessels under the diaphragm, the autonomic system influencing the general vasoconstrictor tone, the skeletal muscles of the lower limb with its “pump function” and the neurohormonal response [21]. The impact of postural change on LVSV can been described as a manifestation of Frank-Starling mechanism, which has been observed in previous studies as an adaptive response to orthostatic stress [, 22, 23]. Clinically this effect is useful in evaluation of fluid response. The passive leg raising (PLR) test has been used to evaluate volume response in various clinical settings. PLR increased stroke volume > 10% in 45% of the healthy subjects [24]. Sureh et al. reported a significant increase in left ventricular volume and SV after PLR in patients with coronary artery disease and cardiac surgery in a prospective study [25]. Further data is available from critically ill patients where PLR increased the enddiastolic volume and cardiac output predicting fluid response [26]. Corresponding to this pathophysiologic concept, LAVi increased significantly in left lateral position demonstrating the left atrial reservoir function [27].

The dependence of LV function - intrinsic contractility and relaxation - on pre- and afterload is an accepted physiological concept [28]. Nafati el al. showed that GLS was affected by different preload conditions in a population of intensive care unit patients [29]. Negishi et al. demonstrated the influence of preload modification on hemodynamic and echocardiographic parameters including GLS by tilt-induced hydrostatic stress emulating different types of gravity in astronauts [30]. Likewise, clinical studies of patients with aortic valve stenosis treated by transcatheter aortic valve implantation or surgical aortic valve replacement, have documented an improvement of GLS due to reduced afterload conditions after therapy [, , 31–33]. Roy et al. reported 60 patients with acute circulatory failure, PLR test - suggesting fluid responsiveness - was positive in 55% of the patients. The VTI_LVOT_ increased according in this group. The longitudinal strain increased by 19%, but the increase in GLS was also shown in the group with negative PLR [34]. This underlines the preload dependence of GLS. Both, LVEF and GLS, are pre- and afterload dependent, but the models of failing hearts suggest that GLS may be more sensitive to preload than LVEF, whereas the latter is more afterload dependent as shown in 1065 patients with heart failure and reduced LVEF [35]. Moreover, the prognostic value of GLS was superior compared with all other echocardiographic parameters. GLS is supposed to be an important parameter to characterize LV function in comparison to LVEF – especially to detect subclinical LV dysfunction. A GLS-reduction of 15% was shown by Locquet et al. in a prospective study in oncologic patients evaluating cardiotoxicity after chemotherapy before clinical symptoms appeared [4]. Meanwhile, GLS is recommended by current guidelines [36]. At rest, upright position caused a 27% relative GLS reduction compared to left lateral position in this cohort of healthy athletes which was likely caused by regional deformation abnormalities of the inferior and/or posterolateral wall.

Changes of LV wall motion in the mid-basal inferior and/or posterolateral segments in upright posture, have already been described by Sasaki et al. in a cohort of healthy volunteers [37]. The authors hypothesized that the observed changes of LV wall motion in upright posture might be influenced by the anatomic position of the heart, particularly by its proximity to the diaphragm and posterior mediastinum [37]. Sakurai et al. has described a pseudo-asynergy of the LV inferior wall in normal subjects explained by close contact of the heart with the diaphragm [38]. Whereas wall motion abnormalities of the LV in supine position are strongly related with cardiovascular events and death, changes of LV wall motion in upright posture seem to be a physiological response to position changing [39]. The combination of both changes, the anatomical position of the heart and the altered preload in upright posture, could be a plausible explanation for these findings. To our knowledge, the effects of upright posture on echocardiographic parameters of LV deformation have not previously been described. Although these findings obviously cannot be interpreted as “subclinical LV dysfunction”, they are clinically important because exercise testing in athletes is often performed in an upright posture, especially during treadmill testing. Lower strain values in upright posture could be by impaired LV filling conditions, because GLS measurements are load dependent. In an experimental animal model an increase in LV afterload induced by aortic banding led to a GLS decrease [40]. The decreased GLS in the upright posture compared to the left lateral position is probably due to orthostasis and has significant effects on LV deformation.

Longitudinal strain and work index have been advocated as more reliable methods in the assessment of LV function and are able to detect subtle abnormalities. GWI is derived and incorporates afterload information adjusting the strain results to a noninvasive measurement of LV pressure. From the physiological point of view, the pressure–volume–area predicts myocardial oxygen consumption and provides a tool to describe coupling of LV mechanical performance to energy use [41]. More recently, Russell et al. introduced a non-invasive analysis of LV pressure-strain-loops [10]. Since GLS is preload dependent, GWI as function of GLS and afterload should depend on preload as well as afterload. This explains the significant increase in GWI in left lateral position as shown in this study. The same pathophysiological mechanisms of increased preload or autotransfusion as seen in GLS are responsible for this mechanism. More preload shifts the LV function to the right on the pressure–volume–curve and increases myocardial work. To our knowledge this is the first study, which examines this strong effect of pre-stretching the LV cavity on GWI in healthy subjects. We can confirm the pathophysiological assumption of preload dependency.

In conclusion both GLS and GWI are significantly preload dependent. When considering using those parameters in the assessment of LV function, the body position of the patient needs to be taking into consideration. Unfortunately, probably the most reports on normal values are derived in left lateral position. Therefore, there is an urgent need for normal values depending on the body position.

## Limitations

In this study, only young and healthy athletes were included. Only TTE examinations of athletes with optimal imaging quality were included, athletes with poor imaging were excluded. Thus, the results are not directly transferable on patients with cardiovascular diseases.

## Conclusion

Upright posture has a significant impact on LV deformation. The reduction of regional longitudinal strain in the inferior and/or posterolateral LV segments are presumably explained by different LV filling conditions based on orthostasis and cardiac interaction with the diaphragm. A reduction of regional strain at rest in upright posture is not necessarily pathological but has a significant impact on investigating athletes, especially in case of treadmill testing.
